# Research on genetic characteristics of corolla pattern traits in *Sinningia speciosa*

**DOI:** 10.3389/fpls.2025.1578931

**Published:** 2025-09-19

**Authors:** YanLing Hao

**Affiliations:** College of Agriculture and Horticulture, Chengdu Agricultural College, Chengdu, Sichuan, China

**Keywords:** *Sinningia speciosa*, complete diallel crosses, corolla pattern, genetic characteristics, traits

## Abstract

**Background:**

*Sinningia speciosa* Benth., one of the earliest commercialized plants in the Gesneriaceae family, is renowned for its rich corolla patterns and vibrant colors. Studying the genetic characteristics of corolla pattern traits in *Sinningia speciosa* will significantly improve breeding efficiency, facilitate the development of more varieties with diverse floral patterns, and provide guidance for the transformation and innovative utilization of Chinese Gesneriaceae resources.

**Methods:**

A comprehensive statistical classification was conducted on 259 accessions from different regions and 1,444 F_1_ hybrids derived from complete diallel crosses of 38 distinctive traits. Further selfing identification was performed on F_2_ progeny, and combining ability and heritability analyses were carried out based on trait classification to determine the genetic characteristics of these traits.

**Results:**

The main popular traits of *Sinningia speciosa* include distinctive petal lobe patterns, unique corolla tubes, throat patterns, and semi-double flowers. Popular petal lobe patterns include Pockmarks, stripes, rainbows, white edges, mottling, and staining. Long-tubed corollas are predominantly labiate, while short-tubed corollas are mainly campanulate. In addition to striped throats, colored throats are also distinctive traits of *Sinningia speciosa*. Selfing progeny analysis revealed that petal patterns such as “Pockmarks” and “stripes,” as well as long corolla tubes, exhibit stable inheritance across successive generations. Complete diallel crosses of F_1_ and F_2_ progeny showed that all distinctive traits followed paternal inheritance, with paternal traits appearing more frequently than maternal traits in F_1_/F_2_ progeny. The highest probability of producing semi-double flowers was observed in single-petal × double-petal crosses. Among the distinctive traits, the general combining ability (GCA) effects were highest for rainbow rings, Pockmarks, and stripes, which are easier to stabilize and fix in early generations. Broad-sense heritability (H²B) and narrow-sense heritability (H²N) were highest for Pockmarks, rainbow rings, staining, and long tubes, suggesting that trait selection should be conducted in early hybrid generations during breeding.

**Conclusion:**

The genetic characteristics of corolla traits in *Sinningia speciosa* are complex and diverse. Breeding plans should be tailored based on the genetic properties of these traits to shorten the breeding cycle.

## 1 Introduction

The genus Sinningia, belonging to the family Gesneriaceae, is a group of flowering plants named after Wilhelm Sinning (1794–1874), a 19th-century German botanist from Bonn (Boggan, 1991). This genus comprises 65 to 70 species, all of which are perennial herbaceous tuberous plants native to Central and South America, with a particular concentration in southern Brazil (Smith, 2019). The corolla pattern, as one of its remarkable features, includes complex phenotypes such as Pockmarks, stripes, veins, and color gradients. It not only has aesthetic value but also contains important information on plant developmental biology and ecological adaptation. Research has revealed that the formation of the Sinningia corolla pattern mainly occurs in the early stages of petal development and is closely related to the spatiotemporal regulation of cell differentiation and pigment synthesis. Multiple genes related to the formation of the corolla pattern have been identified, including genes regulating pigment synthesis (such as CHS, DFR, ANS in the anthocyanin synthesis pathway) and genes regulating cell differentiation (such as transcription factors MYB, bHLH, etc.) (Chen, 2020). Corolla patterns (such as Pockmarks, stripes, etc.) are commonly known as “floral guides” and play an important role in the interaction between plants and pollinators. The formation of the corolla pattern begins in the early stage of petal development. Epidermal cells differentiate into two types: ordinary epidermal cells and specialized pigment cells (such as anthocyanin - accumulating cells). This differentiation is regulated by cell - specific gene expression (Oliveira, 2018). The spot or stripe regions in the Sinningia corolla are closely related to the activity of the MYB-bHLH-WD40 (MBW) complex (Yamaguchi, 2021). The corolla patterns of Paulownia davidiana were classified into five categories, and several candidate genes related to pattern formation were identified by GWAS analysis, the SsMYB1 gene is highly expressed in the spot - forming sites and regulates local pigment deposition by activating anthocyanin synthesis genes (such as CHS, DFR, and ANS) (Zhang, 2021). In addition, transporter genes (such as GST) may be involved in the transport of pigments from the synthesis site to the vacuole, affecting the clarity of the pattern (Fenster, 2004).

Although the process of pattern formation can be inferred from cytological and pigment localization analyses, there has yet to be a systematic summary of how many typical traits currently exist in the corolla patterns of cultivated Sinningia species, what the genetic characteristics of these traits are, how stable their inheritance is, and which distinctive traits can be selected as breeding target selection, how to carry out targeted cultivar breeding based on the genetic characteristics of these traits as so on. Research on the phenotypic characteristics of Sinningia can provide valuable insights and guidance for breeding within the same family or genus with similar phenotypic traits. It holds significant importance for the development and utilization of Gesneriaceae plants in China and for the future industrialization of breeding efforts.

## 2 Materials and methods

### 2.1 Materials and experimental methods

The experiment was conducted continuously for seven years, with all implantation conditions remaining unchanged throughout the period. The breeding materials for both the planted species and native species were provided by Chengdu Agricultural College and Sichuan Agricultural University. A total of 259 breeding materials were collected from 11 countries and regions worldwide, each exhibiting distinct typical characteristics. Based on petal traits, the test materials were categorized according to corolla characteristics, and three representative samples from each category were selected, resulting in a total of 114 typical materials. These materials were subjected to a complete diallel cross, repeated three times. The probability of trait expression in the offspring was investigated, and selected individuals from each category were continuously selfed for seven generations to observe their genetic characteristics.

### 2.2 Measurement methods

#### 2.2.1 Petal and petal lobe trait descriptions

The front color of petal lobes was categorized into primary and secondary colors, along with descriptions of stripes, Pockmarks, and special edge colors on the lobes. Distinctive traits such as white edges, the size of rainbow rings, and the density and clarity of stripes and Pockmarks were recorded (Li and Wang, 2005).

#### 2.2.2 Corolla tube traits

The lower fused tubular part of the corolla is referred to as the corolla tube. In this experiment, the observation focused on the length from the junction of the corolla lobes and the corolla tube to the base of the calyx. Corolla tubes ≥5 cm were classified as long tubes, while those<5 cm were considered short tubes. The corolla tubes of *Sinningia speciosa* are categorized into funnel-shaped, bell-shaped, and lip-shaped types. The flower posture is determined by the angle between the pedicel and the corolla tube, including side-facing (90°), downward-facing (−30° to 45°), and upward-facing (0°) types (Zhang, 2021).

#### 2.2.3 Measurement of genetic traits in hybrid progeny

The semi-double flower series was classified based on literature, with the number of segregating progeny recorded. The length of the corolla tube was measured using a vernier caliper, from the base of the calyx to the horizontal length of the corolla limb. Other characteristic traits were described according to current popular descriptions of *Sinningia speciosa* (Hsu et al., 2017).

#### 2.2.4 Combining ability analysis

Combining ability analysis was conducted using the complete diallel cross method introduced by Liu Laifu (1984) (Liu et al., 1984) General combining ability (GCA) was calculated as g_i = x_i - X_{.}*gi*​=*xi*​−*X*.​, and special combining ability (SCA) was calculated as S_{ij} = x_{ij} - X_{.} - g_i - g_j*Sij*​=*xij*​−*X*.​−*gi*​−*gj*​. Here, x_i*xi*​ and x_j*xj*​ represent the mean trait values of the crosses involving the i*i*-th and j*j*-th parents, respectively, and X_{.}*X*.​ is the overall mean of all crosses. The Chi-square distance was calculated after normalizing the trait values and GCA of the 11 parental lines, and cluster analysis was performed using the sum of squared deviations. Data statistics and processing were completed using Excel 2003 and DPS 7.05 software.

#### 2.2.5 Heritability analysis


$$\begin{array}{c} \text{Broad-sense heritability}\\ =\left(\text{genetic variance}/\text{phenotypic variance}\right)\times 100\%; \end{array}$$

$$\begin{array}{c} \text{Narrow-sense heritability}\\ =\left(\text{additive genetic variance}/\text{phenotypic variance}\right)\times 100\%. \end{array}$$

### 2.3 Data processing

The average values of the experimental results over six consecutive years were calculated. Data processing was performed using SPSS 17.0, and differences were compared using the least significant difference (LSD) method at a significance level of P = 0.05P=0.05.

## 3 Results and analysis

### 3.1 Construction of genetic populations for distinctive traits in *Sinningia speciosa*

#### 3.1.1 Example analysis of corolla characteristics in trait-specific populations

**Figure 1:**
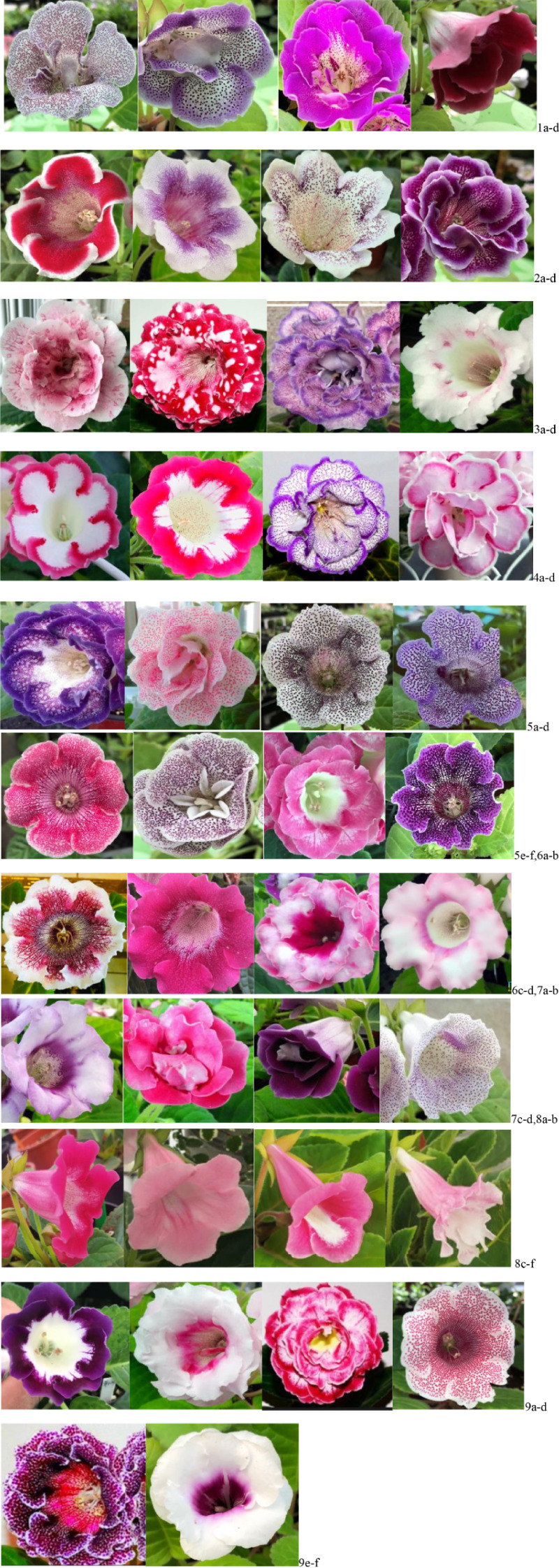
Illustration of actual specimens showing the distinctive characteristics of the Saintpaulia corolla.

**Figure 2:**
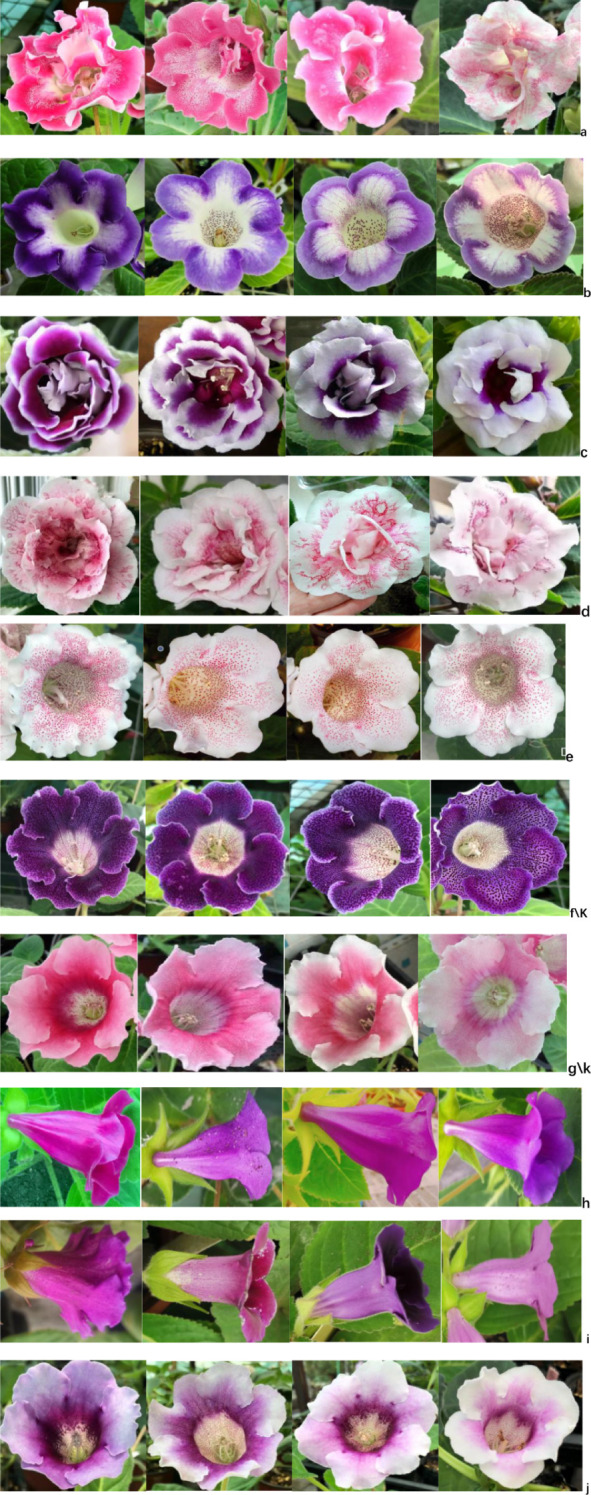
Corolla characteristics on inbred progeny genetic expression. **(a)** HDP **(b)** RR **(c)** WE **(d)** PM **(e)** MO **(f/k)** S + ST **(g/k)** SM + ST **(h)** LLCT **(i)** SLCT **(j)** CT SLCT **(j)** CT.

**Figure 3:**
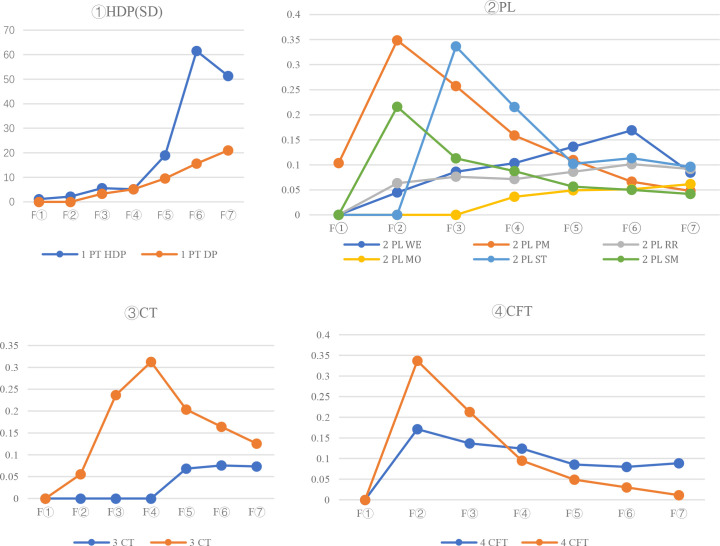
Analysis of the change curve of characteristic traits in the process of continuous self-crossing.

**Figure 4:**
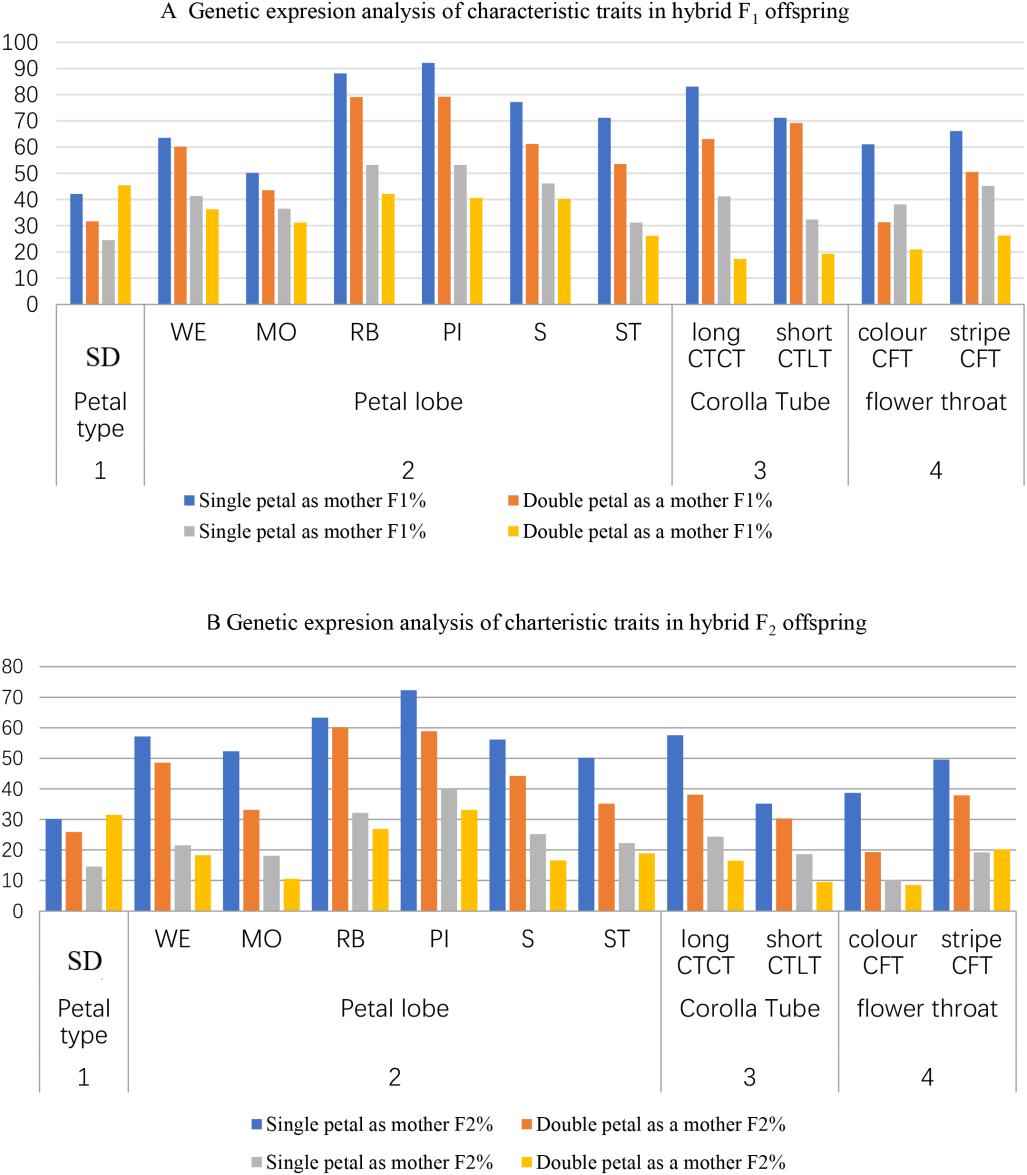
**(A)** Genetic expresion analysis of characteristic traits in hybrid F_1_ offspring. **(B)** Genetic expresion analysis of charteristic traits in hybrid F_2_ offspring .

The comprehensive trait characteristics of *Sinningia* sp*eciosa* with different corolla types were classified into 11 categories. Representative examples of distinctive traits are illustrated in **Figure 1 (1-9)**, including semi-double flowers, unique petal lobe traits (white-edged, rainbow-edged, stained, spotted, striped, and mottled patterns), specialized corolla tubes, and distinctive throat patterns in both single-petal and double-petal progeny. ①Semi-double or Half double petal: The number of petal layers is between 1.5-2. (**Figure 1-1**) ②White-edged trait: Refers to petal lobes with variable-width white margins (**Figure 1-2**). ③Mottled trait: Characterized by irregularly distributed patches of contrasting colors on the petal lobes (**Figure 1-3**). ④ Rainbow-Rings trait: Features concentric color rings along the petal margins, with darker hues forming closed annular patterns (**Figure 1-4**). ⑤Pockmarks trait: Exhibits colored dots of varying sizes and densities, which may either dominate the petal lobe coloration or contrast with a base color (**Figure 1-5**). ⑥Striped trait: Displays one or multiple longitudinal stripes on the petal lobes (**Figure 1-6**). ⑦Smudge(Staining) trait: Shows gradient-like color permeation across petal lobes, resembling dye diffusion (**Figure 1-7**). In current horticultural trends, upward-facing flowers (0° orientation) dominate in *Sinningia* sp*eciosa* (Hsu et al., 2015). However, this study focuses on specialized corolla tubes in side-facing (90°) and downward-facing (−30° to 45°) orientations. These tubes are categorized into three types based on morphology: ⑧campanulate, labiate, and funnelform (**Figure 1-8**). Additionally, while typical corolla throats in *Sinningia* sp*eciosa* often exhibit Pockmarks (Busch et al., 2012), ⑨the “distinctive throat patterns” in this experiment include color variations and striped designs (**Figure 1-9**).

**Figure 1** and **Table 1** comprehensively summarize the key corolla traits, including: ①Width of white margins on corolla edges; ②Size and shape of mottled patches on petal lobes; ③Color intensity, margin width, and spatial distribution of rainbow-edged patterns; ④Pockmark color, size, and density on petal lobes; ⑤Stripe length and quantity; ⑥Uniformity of petal lobe coloration; ⑦Corolla tube morphology and petal lobe edge characteristics; ⑧Throat color and unique pattern combinations. These traits form the foundation for the vast phenotypic diversity observed in *Sinningia* sp*eciosa*.

**Table 1:** Phenotypic distribution of F_2_ progeny traits.

C	Category	C	P	Main traits	C	PTSS	Secondary traits	C	SSG
1	Petal type(PT)	11	1.57%	(SD)\HDP	11	1.57%	2 layers	3	1.57%
							1.5 layers	8	
2	Petal lobe (PL)	522	74.46%	WD	44	6.28%	Pink-WD	9	8.43%
							Red-WD	16	
							Blue-WD	4	
							Purple-WD	15	
				MO	35	4.99%	Pink-Mo	12	6.71%
							Red- Mo	12	
							Purple-Mo	6	
							Wine Dens	6	
				RR	128	18.26%	Pink- RR	71	24.52%
							Red- RR	37	
							Blue- RR	7	
							Purple- RR	13	
				PM	217	30.96%	White PM	11	41.57%
							Pink PM	30	
							Black PM	12	
							Red PM	70	
							Blue PM	6	
							Purple PM	88	
				S	65	9.27%	One -ST -diphtheria	9	12.45%
							One- ST	11	
							Three- ST	30	
							Multiple- ST	15	
				SM	33	4.71%	Red-SM	12	6.32%
							Pink-SM	12	
							Purple-SM	6	
							Other-SM	3	
3	Corolla Tube (CT)	61	8.71%	LLT	49	6.99%	Funnelform type	13	80.32%
							Labiate-type	23	
							Companulate-type	13	
				SLT	12	1.71%	Funnelform-type	1	19.67%
							Companulate-type	10	
							Labiate-type	1	
4	Characteristic flower throat (CFT)	107	15.26%	CT	77	10.98%	Diphtheria	26	71.96%
							Pink-CT	10	
							Red-CT	13	
							Yellow-CT	17	
							Purple-CT	11	
				ST	30	4.28%	Striped-ST	30	28.04%

C, Code; P, percentage; PTSS, Percentage of total sample size; SSG, Share in same group.

#### 3.1.2 Genetic expression analysis of corolla traits in distinctive populations

The characteristics of *Sinningia* sp*eciosa* with different corolla types are compared in **Table 1**. The distinctive traits are primarily manifested in four major categories: 1.Petal Lobe Traits: 522 cases (74.57%), the most prevalent, serving as a key criterion for distinguishing new cultivars;2.Throat Patterns: 107 cases (15.28%);3.Corolla Tube Traits: 61 cases (8.71%);4.Semi-Double Flowers: 11 cases (1.57%).As illustrated in **Figure 1**, the distribution of petal lobe traits is as follows: ①Pockmarks: 217 cases (41.57%), the most numers; ②Rainbow Rings: 128 cases (24.52%); ③Stripes: 98 cases (18.77%); ④White Edges: 44 cases (8.42%); ⑤Mottling: 35 cases (6.70%); ⑥Staining: 33 cases (6.32%). These traits highlight the predominant patterns in *S.* sp*eciosa* petal lobes. Among throat patterns, colored throats (excluding stripes, which account for 28.03%) are also a distinctive feature.

#### 3.1.3 Genetic expression analysis of corolla traits in distinctive populations

The characteristics of *Sinningia* sp*eciosa* with different corolla types are compared in **Table 1**. The distinctive traits are primarily manifested in four major categories: Regarding corolla tubes, the ratio of long to short tubes is 80.32%:19.67%. Long tubes are predominantly labiate (23 cases, 54.09%), while short tubes are mainly campanulate (10 cases, 16.39%). funnelform and labiate tubes each account for 1.64%. Additionally, semi-double flowers (11 cases, 1.57%) are currently a popular floral type.

### 3.2 Genetic expression analysis of self-crossed and hybrid offspring of Anthurium

#### 3.2.1 Genetic expression analysis of self-crossed and hybrid offspring of Anthurium

##### 3.2.1.1 Analysis of variation curves of parameters of characteristic corolla type traits in self-bred progeny

The performance of petal lobe traits in different corolla types after seven generations of selfing is illustrated in **Figures 2** and **3** presents the segregation dynamics of these traits during continuous selfing (generations F_1_–F_7_): These traits can be broadly categorized into three groups, Stable Traits: Pockmarks (PM) and stripes (ST) on petal lobes, along with long lateral corolla tubes (LLCT), showed minimal phenotypic changes across generations. Moderately Variable Traits: Rainbow rings (RR) and white edges (WE) exhibited noticeable changes in area and color intensity, though their core characteristics remained identifiable. Highly Variable Traits: Semi-double flowers (HDP) and mottling (MO) displayed significant alterations, including spot fading and reduced color saturation.

##### 3.2.1.2 Genetic expression in F_1_-F_2_ hybrids

Complete diallel crosses of parents with distinct traits revealed following patterns (**Figures 4A, B**):

1. Paternal Inheritance Dominance: All traits appeared more frequently when the paternal parent carried the trait.
2. Semi-double flowers were most prevalent in single-petal × double-petal crosses (F_1_: 42.15–45.44%; F_2_: 30.13–31.56%).
3. Petal lobe traits like Pockmarks (F_2_: 72.31% paternal *vs*. 58.92% maternal), rainbow rings (63.28% *vs*. 60.23%), and stripes (56.13% *vs*. 44.26%) showed higher paternal transmission.
4. Corolla Tube Stability: Long lateral tubes (F_2_: 24.33% paternal, 16.55% maternal) were more stable than short tubes (F_2_: 18.65% paternal, 9.57% maternal).
5. Throat Patterns: Striped throats (F_2_: 19.23–20.23%) were inherited more reliably than colored throats (F_2_: 10.22–8.49%).

Low-Frequency Traits: Mottling (18.13–10.56%), staining (22.23–18.89%), and colored throats exhibited reduced expression in F_2_ progeny.

#### 3.2.2 Combining ability and heritability analysis of corolla traits in hybrid progeny

General Combining Ability (GCA) reflects the average performance of a parent in a set of hybrid combinations, serving as a critical indicator of breeding potential. Its magnitude and sign indicate the degree and direction of additive gene effects (Blinstrubien et al., 2020). As shown in **Table 2A**, **Table 2B**, paternal GCA values for all 11 traits across four categories are higher than maternal GCA values, a unique hybridization pattern distinguishing *Sinningia* sp*eciosa* from other ornamental plants. Paternal GCA: The highest values were observed for rainbow rings (51.3739), Pockmarks (75.5624), and stripes (46.6604), followed by staining (24.1184). White edges (-26.3553) and long lateral tubes (-33.4259) exhibited the most negative effects. Maternal GCA: The highest values were recorded for rainbow rings (28.623) and Pockmarks (38.852), with decreasing values for long lateral tubes (16.2138), staining (16.8715), and stripes (15.325). Notably, white edges (-46.539) and colored throats (-23.501) showed strong negative effects. Combined with **Figure 2**, these results suggest that long lateral tubes and Pockmarks are controlled by major genes and exhibit stable inheritance. In contrast, stripes and rainbow rings are influenced by both additive and non-additive gene effects, leading to phenotypic variability. Traits such as white edges, colored throats, and long lateral tubes display the highest negative additive effects, necessitating extended selection cycles to stabilize these traits in progeny.

**Table 2A:** Analysis of genotype variance and heritability for paternal traits in tested varieties/lines.

C	Type	MC	CFEV (AV)			Error	GCAV (AV)		VG\VS	Heritability%	
			GCA		SCA		GCA	SCA		H²B	H²N
			♂P1	♀Px							
1	PT	HDP(SD)	16.371	13.1613	-7.4135	2.3721	51.87	68.63	0.7558	81.38	49.5
2	PL	WE	-26.3553	-7.4135	-3.4144	0. 159	24.82	79.2	0.3134	91.32	39.51
		MO	-17.0229	-3.372	23.8619	1.1629	22.36	77.77	0.2875	67.35	17.78
		RB	61.3739	11.0794	29.5784	0.7764	83.71	36.29	2.3067	95.23	68.03
		PM	75.5624	9.7404	31.1306	20.29	78.29	41.55	1.8842	96.91	75.87
		SM	46.6604	18.9464	11.0794	0.3532	53.83	38.57	1.3956	52.62	39.56
		S	44.1184	17.5784	19.4964	2.4007	50.25	74.75	0.6767	85.6	68.65
3	CT	LLCT	-33.4259	15.4964	13.1613	0. 154	63.89	26.1	2.4479	99.33	64.26
		SLCT	13.5121	19.222	-9.7404	0.006	57.83	62. 17	0.9302	79.03	12.66
4	CFT	CT	-18.9464	3.8619	-11.6999	1.121	13.68	81.25	0.1684	65.18	5.71
		ST	16.371	11.6999	13.9613	0.265	8.75	46.73	0.1872	77.7	12.44

**Table 2B:** Analysis of genotype variance and heritability for female-parent combining ability of varieties (Lines).

C	Type	MC	CFEV(AV)			Error	GCAV(AV)		VG\VS	Heritability%	
			GCA		SCA		GCA	SCA		H²B	H²N
			♂Px	♀P2							
1	PT	HDP(SD)	3.5464	-5.8096	-5.8215	0.7141	71.73	78.63	0.8823	77.38	11.75
2	PL	WE	12.875	-46.539	0.7673	1.7589	54.22	45.78	1.1844	82.92	29.45
		MO	5.8977	-3.0452	16.191	2.0318	53.66	47.23	0.5127	67.85	9.78
		RB	15.6639	28.623	21.326	1.0928	73.21	26.79	2.7327	83.69	50.71
		PM	41.0112	38.852	30. 993	0.8238	78.29	21.75	3.5995	88.31	56.87
		SM	16.1399	25.325	9. 993	1.2354	91.83	8.17	11.2399	52.62	31.56
		S	3.253	16.8715	17. 4018	1.8841	15.55	84.45	0.1841	86.86	43.19
3	CT	LLCT	11.13	-28.2138	2. 5389	0.9482	33.89	66.1	1.1361	96.83	34.57
		SLCT	13.512	9.222	22. 932	0.0519	17.83	82. 17	0.2169	34.67	7.36
4	CFT	CT	3.012	-23.501	-11.5012	0.4948	3.6	91.25	0.0395	44.37	5.71
		ST	10.523	8.154	5.236	1.5653	8.75	96.43	0.0907	60.32	9.84

MC, Mian Character; CFEV, Combining force effect value;GCAV, General Combining ability Variance.

It evaluates the extent to which a particular parent deviates from its average performance in hybrid progeny. SCA arises from non-additive genetic effects, is expressed only in hybrids, and is not stably inherited across generations (Moazzeni et al., 2020). The results in this study serve as a comprehensive reference for parental trait selection. The consistency of these findings is further supported by **Figures 2**, **3**.

Heritability reflects the proportion of genetic variance to phenotypic variance, indicating the degree to which parental traits are transmitted to their offspring. It is a crucial quantitative parameter in genetics and breeding, representing the relative importance of genetic determination versus environmental influence in the inheritance of quantitative traits (Rind et al., 2023). Based on the performance of 21 traits in incomplete diallel crosses (**Table 2A**, **Table 2B**, the narrow-sense heritability (H²N) of paternal traits is higher than that of maternal traits. Traits with high broad-sense heritability (H²B), such as long lateral tubes, Pockmarks, rainbow rings, white edges, and stripes, rank highly in narrow-sense heritability (H²N), with Pockmark, rainbow rings, staining, and long lateral tubes being the top four. These traits are less influenced by environmental factors and exhibit strong additive genetic effects, making them suitable for early-generation selection. Traits like “rainbow rings,” “ Pockmarks,” and “stripes” show high H²B and H²N, with genetic variance (Vg) greater than environmental variance (Vs), indicating that additive effects dominate their inheritance. In contrast, other traits with low H²B and H²N, where Vg< Vs, suggest significant dominance and epistatic effects, necessitating extended selection cycles.

## 4 Discussion

The common corolla types of *Sinningia speciosa* are campanulate, with throats often displaying Pockmarks of varying sizes and vibrant flower colors (Chae et al., 2012). This study systematically analyzed 259 *Sinningia speciosa* accessions from different sources and 38 characteristic traits through complete diallel crossbreeding, revealing the genetic characteristics of corolla pattern traits. The results not only provide important theoretical foundations for *Sinningia speciosa* breeding but also uncover innovative genetic patterns, offering significant insights for optimizing breeding strategies. The diverse and distinctive corolla patterns observed highlight the genetic uniqueness and representativeness of the Sinningia genus (Dong et al., 2018).

This study found that the corolla pattern traits of *Sinningia speciosa* mainly include Pockmarks, stripes, rainbow rings, white edges, mottling, and staining. Among these, Pockmarks and stripes exhibited high stability in successive self-pollinated generations, consistent with the findings of (Zaitlin 2024; Li et al., 2013), suggesting that these traits may be controlled by a few major genes and are easier to fix in breeding. Additionally, the length of the corolla tube is closely related to corolla type: labiate corollas typically have long tubes, while campanulate corollas predominantly have short tubes. This association indicates a potential linkage between corolla tube length and the genetic regulation of corolla type, providing a new direction for further research into the genetic mechanisms of *Sinningia speciosa*. The study also revealed that all characteristic traits in the F_1_ and F_2_ generations exhibited paternal inheritance, meaning that paternal traits appeared more frequently in offspring than maternal traits. This finding contrasts with the results of Li et al. (2013) (Li et al., 2016), who suggested that *Sinningia speciosa* traits are primarily co-dominant or maternally inherited. The innovative discovery of paternal inheritance indicates that trait inheritance in *Sinningia speciosa* may be influenced by differences in parental gene expression or epigenetic regulation, offering a new perspective for future genetic research.

Combining the results of **Figures 4A, B** and **Table 2A**, **Table 2B**, it was concluded that the general combining ability (GCA) of paternal parents is higher than that of maternal parents, reflecting the paternal inheritance characteristic. This phenomenon may be attributed to cytoplasmic inheritance involving plastids and mitochondria (Schneider et al., 2015). When the paternal parent involves two or more pairs of relative traits, non-allelic genes on non-homologous chromosomes undergo free recombination. When these pollen grains combine with maternal gametes, they produce offspring with diverse genotypes and phenotypes, providing a genetic basis for floral diversity (Jong and Shmida, 2024). Some studies have also documented that, although rare, paternal mitochondrial DNA (mtDNA) can integrate into fertilized eggs at very low frequencies, potentially influencing offspring (Ureshino et al., 2015). Additionally, epigenetic changes in paternal parents can also affect offspring (Herman et al., 2012). In summary, the genetic expression of characteristic traits is influenced by multiple factors, resulting in complex diversity (Zaitlin, 2012). The experimental results directly demonstrate the universality of paternal inheritance and the stability of certain traits in *Sinningia speciosa*.

In all F1 and F2 hybrid generations, the probability and number of specific traits appearing in offspring were higher when the same material was used as the paternal parent rather than the maternal parent (**Figures 3(1-4)**). Through successive generations of self-pollination, it was observed that traits such as “ Pockmarks,” “stripes,” and “long tubes” in petal lobes exhibited high stability, indicating their ability to be stably inherited with slow trait degradation. This suggests that these traits are likely controlled by major genes and may result from interactions between cytoplasmic and nuclear DNA (Jong and Shmida, 2024).

Combining the heritability and combining ability analyses from **Figures 4A, B** and **Table 2A**, **Table 2B**, it was found that among the 11 characteristic traits of *Sinningia speciosa*, the more stable traits—” Pockmarks,” “stripes,” “rainbow rings,” and “long tubes”—exhibited high general combining ability (GCA) as parental traits. These traits appeared frequently in F_1_ and F_2_ generations with a higher number of offspring displaying the traits, indicating strong additive effects of alleles and non-alleles that can be stably inherited. This highlights the strong genetic characteristics of *Sinningia speciosa* corolla patterns, providing valuable references for future breeding selection. For traits with high GCA, early-generation selection can be implemented. The key to improving breeding efficiency and success lies in the flexible selection of parental trait combinations based on breeding objectives. The experiment aimed to verify the combining ability of specific trait categories in parents and did not fully reflect the specific combining ability (SCA) of individual traits. Therefore, a more detailed analysis is needed to elucidate the SCA of specific parental combinations. However, the results indirectly indicate that traits such as “ Pockmarks,” “stripes,” and “rainbow rings” also exhibit high SCA, with certain parental combinations making special contributions to offspring traits (Zaitlin, 2012).

In this study, petal lobe traits such as “Pockmarks,” “rainbow rings,” “staining,” and “long tubes” showed high broad-sense heritability (H²B) and narrow-sense heritability (H²N), indicating that these traits are primarily controlled by additive gene effects and are strongly influenced by genetic factors. These traits can be stably inherited, and desirable phenotypes can be obtained more quickly through selective breeding, making them valuable for early-generation selection. In contrast, traits such as “white edges” and “colored throats” exhibited high broad-sense heritability but low narrow-sense heritability, suggesting smaller additive genetic effects and larger dominant and epistatic effects. Therefore, it is advisable to increase the number of selection generations for these traits (Perret et al., 2003). Further research is needed to confirm whether epistatic gene interactions contribute to these traits and to elucidate their underlying mechanisms.

## 5 Conclusion

This study systematically revealed, the genetic patterns of corolla pattern traits in *Sinningia speciosa* for the first time, particularly the stability of traits such as Pockmarks and stripes, as well as the phenomenon of paternal inheritance. The discovery of the association between corolla tube length and corolla type provides a new direction for research into the genetic mechanisms of *Sinningia speciosa*. Through combining ability and heritability analysis, the genetic advantages of traits such as rainbow rings and Pockmarks were clarified, offering a scientific basis for early selection in breeding. The study also uncovered the genetic complexity of semi-double petal traits, laying the foundation for cultivating *Sinningia speciosa* varieties with diverse flower forms. In summary, the genetic characteristics of *Sinningia speciosa* corolla traits are complex and diverse. In practical breeding work, breeding plans should be formulated based on these genetic traits to shorten the breeding cycle and improve efficiency. The findings of this study provide important theoretical and practical guidance for the genetic improvement and varietal innovation of *Sinningia speciosa*.

## Data Availability

The datasets presented in this study can be found in online repositories. The names of the repository/repositories and accession number(s) can be found in the article/supplementary material.
